# Electrochemical grippers based on the tuning of surface forces for applications in micro- and nanorobotics

**DOI:** 10.1038/s41598-023-33654-6

**Published:** 2023-05-16

**Authors:** A. Karg, V. Kuznetsov, N. Helfricht, M. Lippitz, G. Papastavrou

**Affiliations:** 1 Physical Chemistry II, University of Bayreuth, Universitätsstraße 30, 95440 Bayreuth, Germany; 2 Experimental Physics III, University of Bayreuth, Universitätsstraße 30, 95440 Bayreuth, Germany

**Keywords:** Atomic force microscopy, Materials science, Electrochemistry, Scanning probe microscopy, Surface assembly

## Abstract

Existing approaches to robotic manipulation often rely on external mechanical devices, such as hydraulic and pneumatic devices or grippers. Both types of devices can be adapted to microrobots only with difficulties and for nanorobots not all. Here, we present a fundamentally different approach that is based on tuning the acting surface forces themselves rather than applying external forces by grippers. Tuning of forces is achieved by the electrochemical control of an electrode’s diffuse layer. Such electrochemical grippers can be integrated directly into an atomic force microscope, allowing for ‘pick and place’ procedures typically used in macroscopic robotics. Due to the low potentials involved, small autonomous robots could as well be equipped with these electrochemical grippers that will be particularly useful in soft robotics as well as nanorobotics. Moreover, these grippers have no moving parts and can be incorporated in new concepts for actuators. The concept can easily be scaled down and applied to a wide range of objects, such as colloids, proteins, and macromolecules.

## Introduction

**Figure 1:**
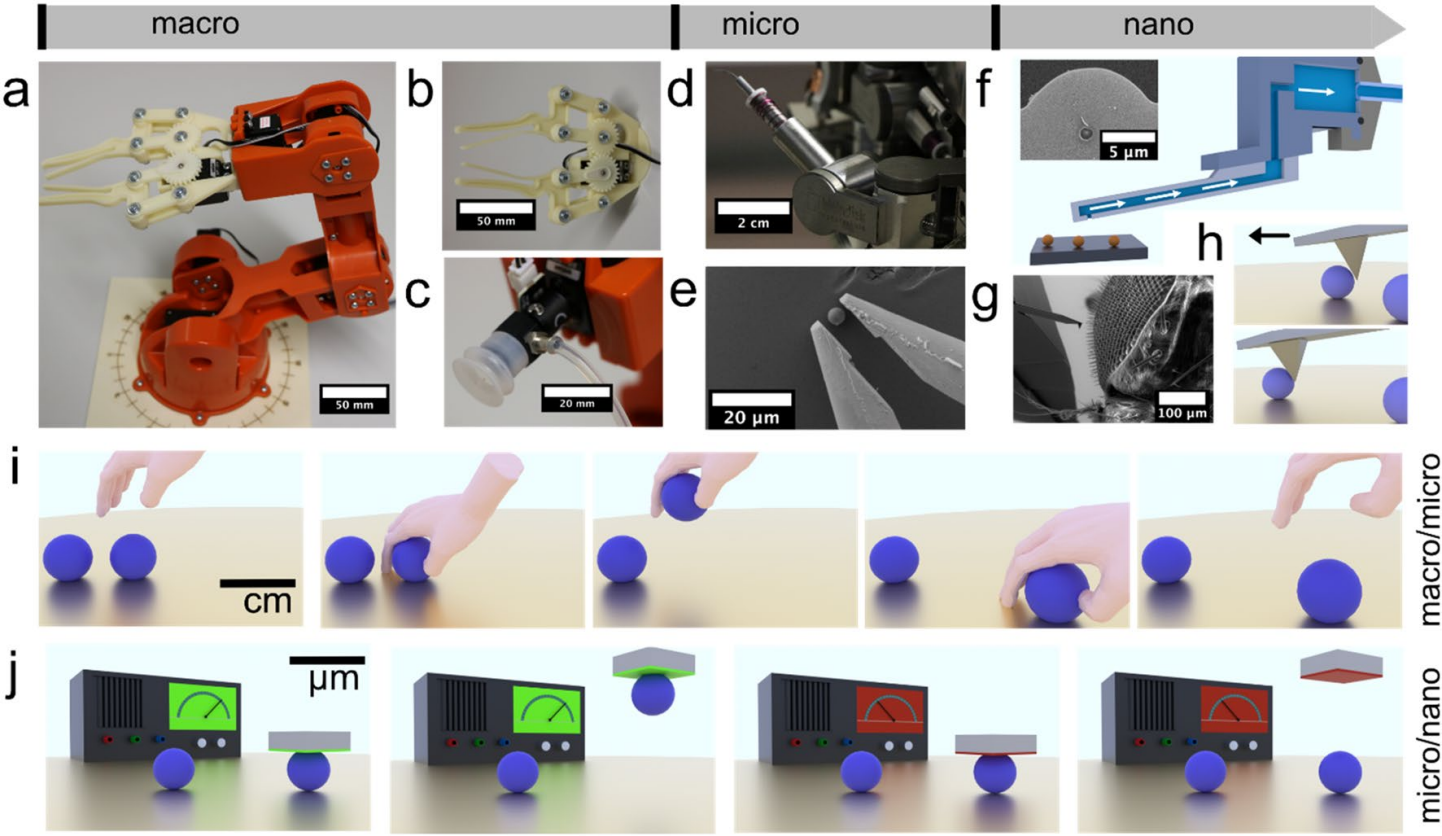
Robotic manipulation principles from macro- to nanoscale. (**a**) Macroscopic 6-axis robot in ‘classic’ design. (**b**) Gripper attachment for the macroscopic robot and (**c**) suction cap**,** respectively. (**d**) An analogous robotic platform for micromanipulation (here, in combination with a scanning electron microscope, SEM). (**e**) Gripper for the aforementioned micromanipulation platform, which allows for the handling of colloidal particles. (**f**) An equivalent of a suction cup that can be combined with an atomic force microscope (AFM). The inset shows a microfluidic hollow AFM cantilever with an aperture of 2 μm in diameter that can be directly connected to a nanofluidic controller. (**g**) The tip of an AFM-cantilever in comparison to the eye of a fly in SEM. (**h**) Example of nanomanipulation by applying shear forces by AFM to move particles to defined places on the sample. (**i**) Single manipulation steps for ‘pick’, ‘place’, and ‘release’, respectively, are illustrated by a human hand on the macroscopic scale. (**j**) Extending the ‘pick’ and ‘place’ concept to the colloidal domain and beyond: rather than applying mechanical pressure the interaction forces are tuned externally. Green indicates attractive interactions (i.e., equivalent to ‘grip’), and red indicates repulsive interactions (i.e., equivalent to ‘release’).

Robotics is a key technology for the twenty-first century. Currently, robots handle objects at length scales from meters down to a few micrometers. Decreasing the length scales, which are routinely accessible by robotic approaches would be of great importance for nanotechnology and medicine. To these means, various micro- and nanorobotic approaches have been pursued in the past years. When reaching the colloidal domain, i.e., few micrometers and smaller, surface forces are starting to become increasingly more important for robotics and well-established concepts of the macroscopic world cannot be applied anymore^1–8^. In particular, the process of ‘pick and place’, i.e., the complex process of gripping, picking-up, and subsequently releasing an object at a defined position becomes more and more difficult to implement^9,10^. Due to the ubiquitous attractive van der Waals (vdW) and capillary forces^1,11^, small objects adhere irreversibly to surfaces. Thus, grippers (cf. Fig. 1a,b), a tool common to macroscopic robotics, become severely limited in their function at small lengths, even when equipped with specifically designed surface modifications ^11–13^. Despite recent advances in the development of novel actuator systems^14,15^ that would allow in principle for a further miniaturization of grippers, the physical limits imposed by surface forces will remain in place. The introduction of novel approaches that rely on manipulating the surface forces themselves rather than optimizing tools from the macroscopic world represents an important step to extend robotic manipulation processes to the low micro- and nanometer scale. Thereby, it will be possible to preserve established manipulation processes like ‘pick and place’ for handling colloidal particles and macromolecules.

For the manipulation of micrometer-sized objects by means of grippers, approaches like increasing the surface roughness, and chemical surface modification of the gripper surfaces have been reported while the manipulation process itself can be followed by light microscopy or scanning electron microscopy (SEM)^16^. The necessity for surface modifications illustrates the increasing influence of surface forces, such as van der Waals and capillary forces, respectively, at decreasing length scales (cf. Fig. 1d,e)^17,18^. Without specifically designed surfaces, objects can be ‘picked’ and ‘placed’ but not subsequently released. Atomic force microscopy (AFM), where a sharp tip is attached to the end of a cantilever (cf. Fig. 1g), is not only a tool to image but also to manipulate objects on the micro- and nanoscale. The field of AFM-based nanorobotics progressed significantly in the last 20 years^5,19,20^. However, the dominating approach of applying lateral forces has remained largely unchallenged (Fig. 1h) and is by far the most-used technique for nanomanipulation by AFM. Nevertheless, by combining AFM with nanofluidics^21,22^, often also referred to as FluidFM-technique, a microscopic analog to suction caps (cf. Fig. 1c) became available at the colloidal scale. This technique allows to handle colloidal objects as small as 300 nm under force control, which allows to directly measure interaction forces (cf. Fig. 1f)^23^. However, a major disadvantage of this technique lies in the fact that microchanneled cantilevers in combination with an external pressure controller are required that do not allow for miniaturized autonomous robots.

Here, we propose a novel approach for micro- and nanorobotic manipulation in liquid environment that is based on externally tuning the interaction forces rather than utilizing miniaturized tools such as grippers and suction caps (cf. Fig. 1b,c), or applying shear forces, respectively. Thus, the ‘pick and place’ process (cf. Fig. 1a) is based on controlling the surface forces themselves rather than exerting ‘external’ forces due to conventional grippers. The sequence in Fig. 1i illustrates the analogy to the manipulation process with grippers (or our hands): Instead of gripping an object one ‘switches on’ a strong attractive interaction force (cf. Fig. 1j), which is still applied during lifting and transferring. The object is released by ‘switching-off’ the strong attractive force and switching subsequently to a less strong interaction than acting between object and substrate. Tuning the adhesion force between an AFM-cantilever and a colloidal object provides thus a direct approach for the manipulation at colloidal length scales without elaborate mechanical devices. Previously, a small number of micromanipulation techniques have been reported based on electric fields in gaseous atmosphere^24,25^. However, the resulting image charges and required large field strengths render this approach not easy for manipulation in liquids^26^. To the best of our knowledge there are only few examples of micromanipulation by electrochemical control: One approach has been based on switching surface properties of a hydrogel by external potentials^27^. However, only objects with a suitable surface chemistry can be manipulated. Another approach has been reported for metallic objects only^28^. By contrast, a large number of electrochemically based actuation systems have been reported in the past^29–32^.

The interaction of colloidal objects is governed by various types of surface forces^18,33^. Which surface forces in liquid environment would be tunable, and strong enough? Van der Waals forces are ubiquitous but cannot be changed without replacing the materials themselves or the medium, respectively. Moreover, van der Waals forces are rather weak, especially in liquid environments. Capillary forces are only present under ambient conditions and are thus not of relevance here. Solvent exclusion can lead to rather strong adhesion forces^34–36^. However, these forces can only be tuned by changing the surface chemistry, which requires complex coatings and external stimuli such as temperature or light^37,38^. The only remaining force contribution in colloid science results from the overlap of the diffuse layers originating from charged surfaces in electrolyte solutions. The concept of diffuse layers (DLs) originates from electrochemistry and the extension of DLs strongly depends on the electrolyte solution composition and the potential applied to the electrode. Diffuse layer forces are known to influence the adhesion of colloidal particles on electrodes and these forces have been studied previously by the colloidal probe technique based on AFM^39–42^. Colloidal probes are force sensors that are prepared by attaching a single colloidal particle to the end of an AFM cantilever^43–46^. Here, we follow a different approach: By equipping an AFM-cantilever with a suitable, flat electrode, which is connected to an external potentiostat, we convert the AFM-cantilever to an ‘electrochemical gripper’ to handle colloidal objects in liquid environments by a ‘pick and place’ procedure.

## Results

### Combining AFM with electrochemistry

**Figure 2:**
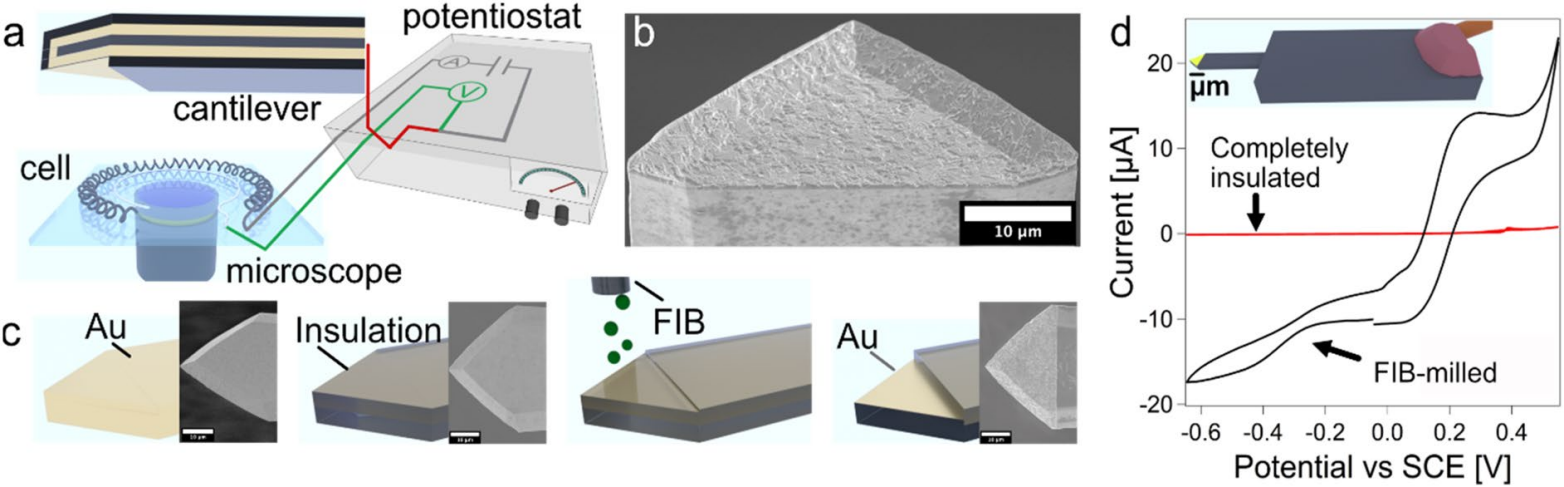
Preparation and characterization of an electrochemical gripper on an AFM-cantilever. (**a**) Schematic representation of the electrochemical setup to control the electrode at the end of an AFM-cantilever by a potentiostat. The electrochemical cell was placed on top of an inverted optical microscope. (**b**) SEM image of a cantilever with an insulation layer, which has been selectively removed at the front. (**c**) Schematic representation of the preparation steps: an insulation layer of electropaint has been deposited onto an AFM-cantilever that has been coated from both sides with gold. Then, this insulation layer has been partially removed from the apex of the cantilever by FIB-milling. (**d**) Cyclic voltammogram (CV) for the thereby formed electrode on the cantilever that acts then as electrochemical gripper. The CV confirms that the electrode is electrochemically active (black) in comparison to a completely insulated cantilever (red).

A proof-of-principle for an AFM-based electrochemical gripper has been given in this study by the manipulation of colloidal silica particles with a diameter of a few microns. This choice of diameter allows to directly visualize the particles by light microscopy while they are still small enough to be dominated in their interaction by surface forces^33^. Figure 2a shows a schematic representation of the experimental setup (cf. also Supplementary Fig. 1): A commercial AFM was mounted on top of an inverted optical light microscope. A purposely made electrochemical cell allows for applying defined potentials to the working electrode, which was here integrated at the apex of a modified AFM-cantilever. Figure 2b shows a scanning electron microscopy (SEM) image of such a purposely made cantilever. These custom-made cantilevers were completely insulated except for their front part, which acted as electrode.

Figure 2c gives an overview on the preparation of these cantilevers, which will be referred to as *electrochemical grippers*. These grippers have been prepared from Au-coated tipless AFM-cantilevers (cf. Fig. 2c, left) that have been electrically contacted by means of thin insulated wires and silver paint. Then, these cantilevers were completely insulated by the deposition of a cathodic electro-paint (cf. Fig. 2c, center left). In the next preparation step, the insulation was removed only at the apex of the cantilever, which represents the later electrode area. The removal has been carried out by focused ion beam (FIB)-milling in the SEM. (Fig. 2c, center right, further details are also given in Supplementary Figs. 2 and 3). In terms of the insulation, this process is similar to the one presented recently for the preparation of electrochemical colloidal probes^47^. In order to confirm that only the front part of the cantilever was electrochemically active (cf. 2c, right), cyclic-voltammetry was performed (cf. Fig. 2d). In a cyclovoltammogram (CV), the applied potential is ramped, and the resulting current is acquired^48^. On macroscopic electrodes with dimensions larger ∼ 25 µm, one finds isolated oxidation and reduction peaks that are specific for an electrochemical redox couple. By contrast for smaller dimensions, i.e., micro- and nanoelectrodes, a sigmoidal shape is expected^48^. Here, the redox couple has been potassium ferrocyanide and potassium ferricyanide^49^. The corresponding peaks for analogous conditions as used in our experiments have been reported to be 0.120 V and 0.240 V (vs SCE), respectively^50^. The electrode area on the apex of the here-prepared cantilever has an area of *A* = 645 µm^2^ (approximation as a triangle). Hence, its critical dimension falls just in the transition region between macro- and micro-electrodes. In consequence, small oxidation and reduction peaks at the expected potentials that are superimposed to an overall sigmoidal shape have been observed (cf. Fig. 2b). In order to verify the insulation properties of the coating, we performed additional experiments acquiring CVs for cantilevers before the FIB-treatment and thus a complete insulation layer. No significant electrochemical activity could be observed in the CV. Further details for the electrochemical experiments (cf. Supplementary Fig. 4) as well as for the preparation of the cantilever (cf. Supplementary Figs. 2 and 3) are given in the SI.

### Interaction forces between colloidal particles and substrates

To establish micromanipulation based on tuning the surface forces in a defined manner, we first had to quantify the acting force contributions. Two sets of interaction forces are of interest here: First, the forces between silica particles and the substrate. The latter were microscopy slides made from borosilicate glass. Second, the forces between the particles and the electrode of the electrochemical gripper. This electrode has been incorporated in an AFM-cantilever (cf. Fig. 2), and its potential has been externally controlled by means of a potentiostat.

**Figure 3:**
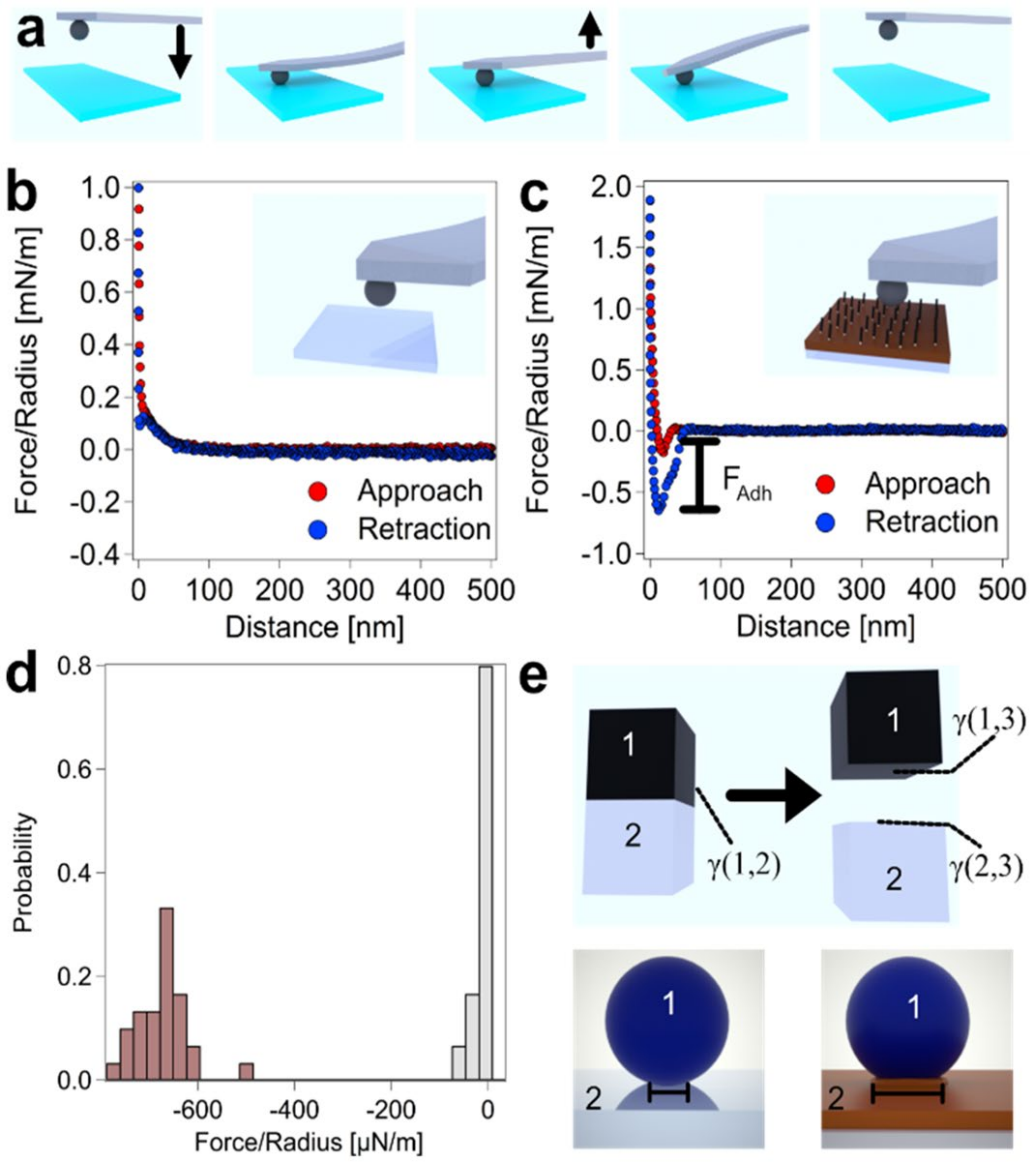
Substrate vs. particle interaction. (**a**) Schematic representation of direct force measurements by conventional colloidal probe AFM in sphere/plane geometry. A silica particle (i.e., the colloidal probe) has been permanently attached to the cantilever and has been approached to the sample surface while the acting force has been sensed simultaneously. Thus, the long-range forces can be determined. Upon reversal of the movement, additionally the adhesive forces (*F*_*Adh*_) can be determined. (**b**) Exemplary force versus distance curve for a silica particle interacting with a bare glass surface in electrolyte solution (pH = 4.0, I = 0.1 mM). No adhesion could be detected in most cases. (**c**) Exemplary force versus distance curve for the interaction of a silica particle with a silane-modified glass surface (contact angle *θ* = 48°) under the same conditions. Here, strong adhesive behavior has been present, which can be attributed to solvent exclusion. (**d**) Distributions of the adhesion forces acquired on both substrates. For each substrate, at least 30 force curves have been measured (**e**) Schematic representation of how solvent exclusion controls the adhesion behavior due to the ‘creation’ and ‘destruction’ of interfaces with the liquid phase. Each interface is by its respective interfacial energy* γ* (top).

Figure 3 shows how the interaction forces between the silica particles and substrates have been determined: We prepared so-called ‘colloidal probes’ by attaching a particle permanently to an AFM-cantilever^43,44^. Such ‘classical’ colloidal probes allow for the acquisition of interaction force profiles between the colloidal particles and the flat substrate in well-defined sphere/plane geometry^51^. The interaction force profiles were acquired by ramping the z-piezo in direction of the substrate and detecting simultaneously the force acting on the colloidal probe as function of the piezo-displacement and separation distance, respectively. A schematic representation of the measurement principle is shown in Fig. 3a and an exemplary force versus distance curve is shown in Fig. 3b.

Starting at large separations, no interaction forces were detectable initially. With decreasing distance, the forces due to the overlap of the diffuse layers started to act. In Fig. 3b the diffuse layer force was repulsive, as expected, since both, the glass surface as well as silica colloid are charged negatively^52,53^. An exponential force law could be recuperated as expected for the diffuse layer overlap. A quantitative analysis of the interaction force profiles based on full solutions of the Poisson-Boltzmann equation including charge regulation is given in the SI (cf. Supplementary Fig. 6). Furthermore, no attractive forces are detected, which can be attributed to the substantial surface roughness on the colloidal particle as well as on the electrode^54,55^. Upon contact of the colloidal probe and the substrate, the interaction is governed additionally by contact mechanics^33,51^. With increasing piezo-displacement the particle was pressed increasingly against the substrate until a pre-defined maximum loading force was reached, where the movement of the z-piezo was reversed. Due to adhesive forces in the contact area, the two surfaces remain in contact. At a certain point, finally a jump-out-of-contact took place when the cantilever’s restoring force overcomes the adhesion forces. Contributions to the adhesion forces are not only given by long-ranged contributions, i.e., diffuse layer overlap and van der Waals forces, but also by contributions within the contact area, such as chemical bonds and solvent exclusion. Stronger adhesion force can be observed on hydrophobized glass substrates, an example is given in Fig. 3c.

**Figure 4:**
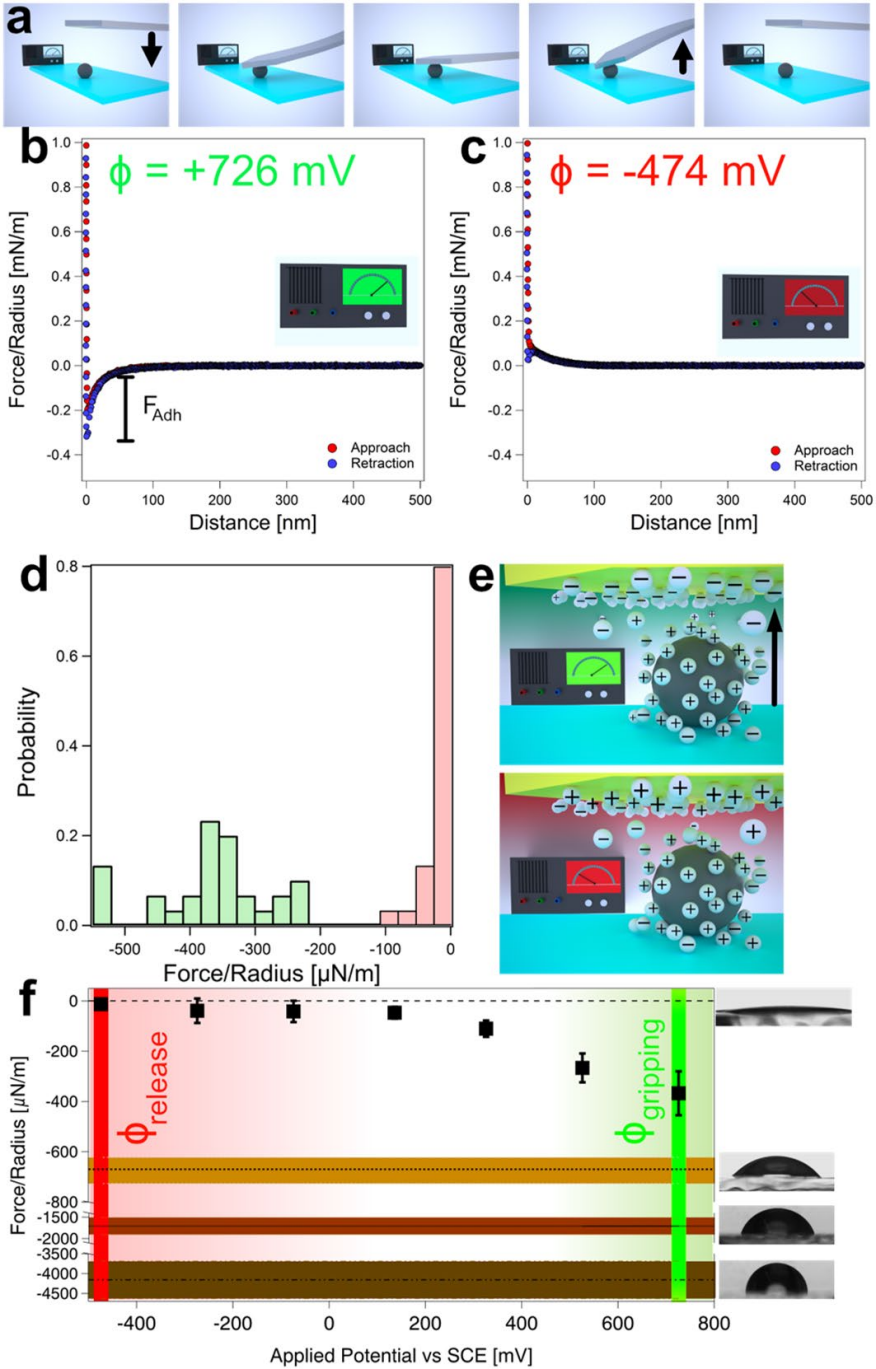
Interaction of the particles with the electrochemical gripper. (**a**) Schematic representation how interaction force profiles between silica particles and the ‘electrochemical gripper’ (i.e., cantilever with an integrated electrode) have been determined: the silica particle has been glued to a flat substrate and remains immobile. (**b**) Exemplary force versus distance curve for an applied potential of *ϕ*_*gripping*_ =  + 726 mV. At this potential the long-range forces are attractive and large adhesion forces can be observed. (**c**) Exemplary force versus distance curve for an applied potential of *ϕ*_*release*_ = − 474 mV. The long-range forces are completely repulsive, and no adhesion can be observed. (**d**) Distribution of adhesion forces *F*_*Adh*_ for *ϕ*_*gripping*_ =  + 726 mV and *ϕ*_*release*_ = − 474 mV. For both potentials, at least 30 force curves have been measured (**e**) Schematic representation of how diffuse layer overlap dominates the interaction force and thus also adhesion of non-hydrophobic electrode surfaces. The overlap of the diffuse ion layers of electrode and particle leads to an osmotic pressure and the resulting force can be repulsive (likewise charged counterions) or attractive (differently charged counterions). (**f**) Adhesion forces as a function of the externally applied potential are summarized. For comparison also, the adhesion forces on the differently modified glass surfaces (*θ* < 15°, *θ* = 48°, *θ* = 77°, and *θ* = 101°) are shown together with images of the corresponding contact angle measurements. The externally applied potential allowed to switch the adhesive behavior from highly adhesive (green, ‘gripping’) to highly repulsive (red, ‘release’).

The interfacial properties of the substrates can be varied in a defined manner by means of gas-phase silanization with methoxy(dimethyl)octylsilane (MDOS)^56,57^, which forms hydrophobic self-assembled monolayers (SAMs). The degree of hydrophobicity has been verified by static measurement of the contact angle *θ* (cf. Supplementary Table 1). Besides the hydrophilic, bare glass surfaces (*θ* < 15°), we studied here the interaction between silica particles and SAMs obtained by gas phase silanization with different exposition times. The resulting contact angles were: *θ* = 48° ± 1°, *θ* = 77° ± 6°, and *θ* = 101° ± 3° (cf. Fig. 4f and Supplementary Table 1), respectively. Figure 3d compares the distribution of the adhesion forces for the exemplary force profiles acquired with a silica colloidal probe and a bare glass surface (cf. Fig. 3b) and a substrate with *θ* = 48° (cf. Fig. 3c). The adhesion force was significantly different for these two substrates. In the case of the silane-modified sample the adhesion forces (*F*_*Adh*_*/R* = 675 ± 52 µN/m) were much larger than for the bare glass surface (*F*_*Adh*_*/R* = 10 ± 19 µN/m). The corresponding adhesion force distributions show a broad distribution. The reasons are manifold but can be mostly attributed to surface roughness and variation on the single molecule level^58–60^. In the framework of the Johnson-Kendall-Roberts (JKR)-theory, which only takes acting forces in the contact area into account, the adhesion force is given by *F*_*Adh*_*/R* = 1.5*πW*_*ad*h_ in the sphere-plane interaction geometry^33^.

The JKR-theory is commonly applied for gold surfaces^34–36^ and has also been utilized here for the interpretation of the adhesion forces due to solvent exclusion only (i.e., without electrostatic contributions due to the potentiostatic control). The work of adhesion *W*_*adh*_ = *γ*_*13*_ + *γ*_*23*_ *− γ*_*12*_ is given by the interfacial energies *γ* of the interfaces created (*γ*_(1,3)_ and *γ*_(2,3)_) and destroyed (*γ*_(1,2)_) (cf. Fig. 3e). The hydrophilicity and surface chemistry of bare glass surfaces and silica particles is similar (*γ*_(1,3)_, *γ*_(2,3)_≈ 1.6 mN/m)^41^ and leads only to small contributions to the adhesion by solvent exclusion. Thus, the interaction of the diffuse layers, which is repulsive and drives the two surfaces away from each other, dominates and leads to no adhesive behavior. The silica particles stick only in a few cases on the glass surface. On the other hand, with additional hydrophobic entities on the glass, the value of *γ*_(1,3)_ increases up to the point where solvent exclusion dominates the diffuse layer repulsion. This finding has been corroborated by the adhesion forces on the substrates with higher contact angles (cf. Fig. 4).

### Interaction forces between colloidal particles and the electrode of the electrochemical gripper

Figure 4a shows in a schematic manner how the interaction forces between the silica colloidal particles and the electrode of the electrochemical gripper have been determined. We introduced a special interaction geometry, in which the colloidal particle has been immobilized by glue on a solid substrate while the potentiostatically controlled cantilever with the electrochemical gripper is positioned directly above the particle. This interaction geometry is practically ‘inverted’ in respect to the conventional sphere/plane configuration present in a colloidal probe (cf. Figs. 3a, 4a). The gold electrode on the cantilever has been modified by a thiol-based SAM terminating in OH-groups in order to render the electrode hydrophilic in a defined manner. Thus, solvent exclusion is not expected to contribute significantly to the adhesion behavior with the modified electrode as for glass and silica, respectively^41^. Figure 4b,c represent two exemplary force profiles for a cantilever under potentiostatic control at different applied potentials. These exemplary force profiles have been selected from the series (n=30) of force versus distance curves acquired for each potential. Figure 4b shows a force profile, which was acquired at a highly positive potential (*ϕ*_*gripping*_ = +726 mV). In this case, the surfaces of the colloid and electrode are oppositely charged. In consequence, the long-ranged electrostatic forces upon approach between the particle and the gripper are attractive. The same is valid for the adhesion forces, where the electrostatic attraction superimposes to the solvent exclusion. By contrast, Fig. 4c shows a force profile acquired at a highly negative applied potential (*ϕ*_*release*_ = − 474 mV). Upon approach the long-range forces are repulsive over the whole distance range, as expected for the interaction between two negatively charged surfaces. Moreover, no adhesion between the surfaces can be detected. Figure 4d shows the corresponding distributions for the adhesion forces at these two applied potentials as determined from all force profiles (n=30) acquired at each potential. Due to the hydrophilic nature of the two surfaces involved, the adhesion forces reflect the long-ranged interaction forces due to the diffuse layer overlap primarily. The latter is also acting upon approach before the two surfaces were in contact. We noticed that no adhesion was taking place for the negative potential, and the interaction remained completely repulsive even when the surfaces were in contact. It should be noted that attractive contributions due to van der Waals forces are strongly reduced due to the surface roughness^58,59^.

The variation of adhesion forces as a function of the external potential has been reported previously, albeit on flat electrodes. It has been probed in sphere/plane geometry by colloidal probes^41,61,62^. We could recently demonstrate that the contribution of the long-range interaction forces due to diffuse layer overlap is essential for the modulation of the adhesion forces^41^. Figure 4e illustrates the influence of the forces due to diffuse layer overlap in a schematic manner and shows how the diffuse layer on the electrode changes in function of the potential applied by the potentiostat: For highly negative potentials, the diffuse layer is composed of cations as counter ions while for highly positive potentials the anions form the counter ions. The diffuse layer decays exponentially from the surface of the electrode until the ion composition reaches the bulk concentration again. At an ionic strength of 0.1 mM, the exponential decay takes place with a Debye-length of 30 nm^33^. The measurements have been performed at pH 4.0, therefore, the silica colloidal particles have a slightly negative diffuse layer charge. Silica as insulator does not change its diffuse layer properties in function of the externally applied potential to the electrode^41,63,64^. The overlap of silica’s diffuse layer with the one of the electrodes leads thus to a repulsive force in the case of a negative potential. (cf. Fig. 4e Bottom). By contrast, a highly positive potential does lead to an attractive interaction force (cf. Fig. 4e Top).

### Particle manipulation by potentiostatic control

Tuning the adhesion in a defined manner by an external signal represents the key to our approach of nanomanipulation by AFM. Figure 4f summarizes the data of adhesion forces for a range of applied potentials (*ϕ* = − 474 mV to *ϕ* = +726 mV vs. SCE). Each data point originates from a distribution analogous to the data shown in Fig. 4d. In the potential range *ϕ* = − 474 mV to +136 mV no adhesion between the particle and the electrode of the gripper has been observed. Due to the lack of adhesive forces, i.e., ‘non-sticking’ of the particle to the electrode, a ‘gripped’ particle would be released to the substrate in this case. To this process, we will refer in the following as ‘*placing*’ a particle. Instead, for potentials *ϕ* > + 136 mV, the adhesion force increased monotonically with increasing the applied potential. Thus, the adhesion force between the particle and the electrode on the gripper becomes larger than the ones between the particle and the substrate. Therefore, these potentials allow for ‘*gripping*’ or ‘*picking*’ a particle from the substrate as the particle will ‘stick’ to the gripper. The transition coincides with the potential of zero charge (pzc), where the electrode is practically uncharged and the long-range forces are minimal^41,64^. For external potentials smaller than the pzc, the diffuse layer interaction is repulsive as particle and electrode are likewise charged. For potentials above the pzc the electrode is reversing its charge to positive. In consequence the long-range forces upon approach are becoming attractive and the adhesion forces are monotonically increasing with increasing the applied potentials. A similar adhesion behavior has been reported previously for studies on flat electrodes with an analogous surface modification^41^. However, a direct comparison of the pzc for the electrodes prepared by FIB and flat electrodes is not possible as the different crystal surfaces of the former are leading to a shift of the pzc^65,66^. In particular, for surfaces subject to a FIB-treatment, this effect is highly pronounced and leads to an increased roughness^67^. A more detailed comparison between the two types of electrodes is given in the SI (cf. Supplementary Fig. 7). The data in Fig. 4f can be divided into a region where the external potentials are leading to a repulsive behavior and thus the ‘placing’ of a particle and a region of potential that corresponds to attractive interaction forces and thus a ‘gripping’ of particles from a substrate.

The ‘*pick and place*’ procedure implemented with the electrochemical gripper makes use of the principle that a particle will transfer most likely to the surface on which a higher adhesion is present. In the following, we illustrate that this process takes place with a high probability, provided that the right potentials are applied to the electrochemical gripper. Thus, if an electrochemical gripper is placed on a particle sitting on a glass substrate, an attractive potential *ϕ* (e.g., *ϕ*_*gripping*_, cf. adhesion force in Fig. 4b in comparison to the one in Fig. 3b) applied to the gripper electrode leads to greater adhesive forces between particle and electrochemical gripper in comparison to the glass. Upon retraction of the cantilever (i.e., the electrochemical gripper), the particle has then been ‘gripped’ (or ‘picked’) as it attaches to the gripper due to the higher adhesive forces. Nonetheless, it can be ‘released’ again to the substrate when a highly negative potential (e.g., *ϕ*_*release*_*)* is applied to the electrode of the electrochemical gripper. In this case, the interaction becomes now more repulsive with the electrode than with the substrate.

**Figure 5:**
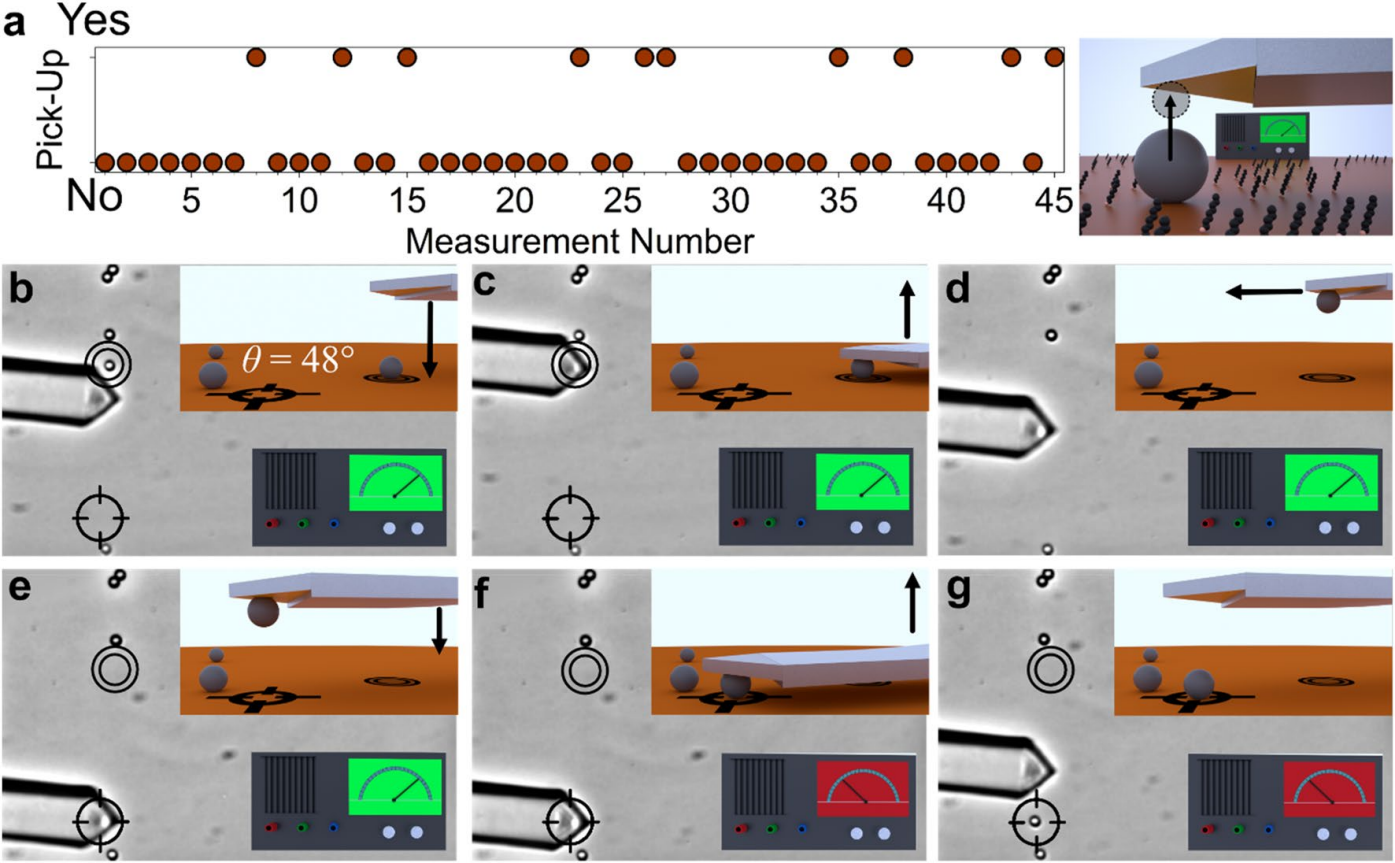
Particle manipulation by an electrochemical gripper. (**a**) Time series for successful events of ‘gripping’ of colloidal silica particles from a silane-modified glass substrate (*θ* = 77°). As shown in the schematic representation on the right, the particle is now not immobilized. A success probability of 0.2 has been observed, while practically all pick-up events have been successful for the bare glass (*θ* < 15°). (**b-g**) Sequence for the different manipulation steps of a single particle by an ‘electrochemical gripper’. The cantilever is set to an attractive ‘gripping’ potential (green) (**b**) and placed over the particle (**c**). Upon retraction of the cantilever the particle remains attached to it (**d**). The cantilever is moved to a new position and placed on the surface (**e**). The interaction is switched to ‘repulsive’ (red) (**f**). Then the cantilever is moved away from the surface and the transferred particle resting on the substrate has been ‘released’ (**g**).

The transfer process by applying *ϕ*_*gripping*_ and subsequently *ϕ*_*release*_ takes place only with a certain probability. The probability depends on the applied potential and the interfacial energy of the substrate, which combined give the total adhesion force. The different parameters influencing the total adhesion forces, in particular the dependence from the externally applied potential (cf. Fig. 4f) has been studied in detail elsewhere, albeit in an inverse geometry^41^. In summary, the the adhesion force depends approximately linearly from the externally applied potential (cf. Fig. 4f) while the interfacial energy (hydrophilic to hydrophobic) leads to an offset in the adhesion forces^41^. In order to demonstrate that highly positive potentials, i.e., of *ϕ*_*gripping*_ = +726 mV vs. SCE, provide a reliable means to remove particles from the substrates, we performed experiments with different substrates at this potential: For bare glass substrate, a transfer of the particle from the substrate to the cantilever was practically always taking place, thus a success rate of *ξ *≈ 1 (n > 30) has been attributed. However, for the slightly hydrophobic silane-modified substrate (*θ* = 77°, cf. Fig. 4f) a success rate of about *ξ* = 0.2 (*n*=45) for successful picking of the particles from the substrate has been observed by optical microscopy. The corresponding sequence is shown in Fig. 5a. Thus, even on hydrophobic substrates, gripping of particles is possible, despite a more unfavorable partition of forces due to solvent exclusion and to diffuse layer overlap.

Figure 5b–f show how the implementation of an electrochemical gripper has been utilized for the defined manipulation of single colloidal particles on a bare glass substrate. The process has been followed by optical microscopy. By applying a high positive potential (*ϕ*_*gripping*_ = +726 mV) to the gripper electrode the adhesion with the particle has been rendered favorable compared to the particle's adhesion with the substrate (cf. Fig. 5b). Consequently, the particle remained on the electrode when the cantilever has been retracted from the substrate (cf. Fig. 5c). Thus, the particle has been ‘gripped’ solely by tuning the surface forces. After being about 10 μm separated from the surface, the cantilever with the gripper was moved to a new position (cf. Fig. 5d) where the cantilever is again approached to the surface (cf. Fig. 5e). A negative potential (*ϕ*_*release*_ = − 474 mV) has been applied (cf. Fig. 5f), which leads to a highly repulsive interaction between the electrochemical gripper and particle. In consequence, the transferred particle is ‘released’ on the substrate on the new position at the substrate upon retraction of the cantilever (cf. Fig. 5g). It must be noted, that the interaction between bare glass and the particle is slightly repulsive as both surfaces are negatively charged. The movement of the electrochemical gripper, i.e., AFM-cantilever, is connected with hydrodynamic movement of the liquid in the vicinity of the particles on the substrate and leads also to slight lateral movements of these particles when the piezoelectric actuator of the AFM has been moved too fast. However, such unintentional lateral movements can be clearly distinguished from the gripping of a particle as in the latter case the particle remains attached to the cantilever.

### Electrochemical gripper for nano- and microrobotics

**Figure 6:**
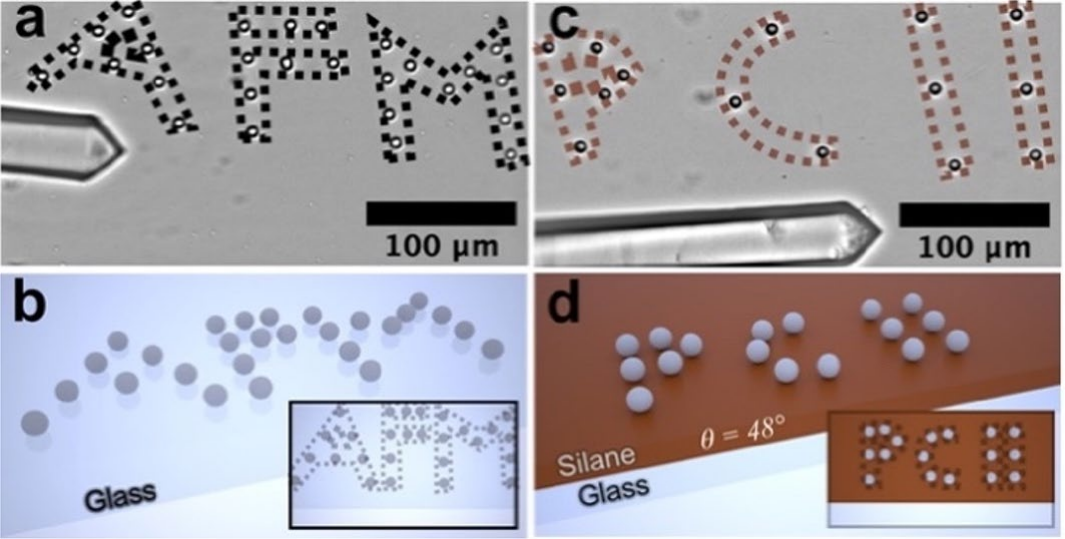
Creating complex structures by electrochemical manipulation. (**a**) Particle structure on a bare glass substrate; the dashed lines serve only to highlight the text ‘AFM’. (**b**) Schematic representation of the structure shown above. (**c**) Another structure but formed on a silane-modified glass slide (*θ* = 48°). The adhesion forces are higher, leading to more stable structures with a lower gripping probability. The abbreviation ‘PC II’ stands for ‘Physical Chemistry II’. (**d**) Schematic representation for structure in (**c**).

Sequential manipulation of single particles allows to perform more complex tasks common in robotics. As proof-of-principle, we prepared the two structures shown in Fig. 6. Our examples of particle arrangements represent the abbreviations ‘AFM’ and ‘PC II’, which stand for ‘atomic force microscopy’ and ‘Physical Chemistry II’. The manipulation processes required about 120 min and 60 min, respectively. To assemble these structures, single particles have been manipulated by the ‘pick and place’ or, more specifically, the ‘gripping and release’ process in a sequential manner. Here, also individual particles were manipulated more than once. A time-lapse movie showing the whole process for writing ‘AFM’ (cf. Fig. 6a) is available in Supplementary Movie 1. We prepared the structures on two different substrates, namely a bare glass slide (‘AFM’, cf. Fig. 6a) and a partially silane-modified glass slide (θ ≈ 48°, PC II, cf. Fig. 6c). The different substrates allowed to demonstrate that manipulation can be performed on a large range of surface chemistries. Additionally, the size of the accessible working area is in this approach only limited by the size of the used liquid cell. The latter substrate has the advantage that the manufactured structures remain much more stable due to the stronger adhesion forces between deposited particles and the substrate.

## Discussion

Tuning surface forces by electrochemistry is a highly universal approach for micro- as well as nanorobotics as it does not require complicated grippers or nanofluidic probes. Most importantly, it can be easily scaled down. The here-presented electrochemical approach can be directly integrated into existing commercial AFMs. Tuning of surface forces by applying potentials is much more direct and faster compared to stimuli-responsive layers that change their surface properties in terms of pH-value or illumination. In particular, the electrochemical grippers can be coupled to a computer in a very direct manner, as most commercial potentiostats can be directly interfaced. Simple electrical signals can then be used as triggers to ‘pick’ and ‘release’ objects. Thereby, it will be easily possible to ‘mimic’ handling algorithms common for macroscopic robotics, in particular ‘pick and place’ (cf. Fig. 1).

The general idea of the electrochemical approach is somehow related to the application of electrostatic forces in air^6,24,38,68^. However, long-ranged electrostatic forces are only working in air or vacuum and requires large potentials and objects with sufficient intrinsic charge. By contrast, the electrochemical gripper represents also an ‘electrical’ approach, but operates with small currents and potentials and, most importantly: Working in liquids, and in particular electrolytes, represents the native environment for this electrochemical approach. As there are no mechanical parts, it is open to miniaturization and does not need external hydraulic or pneumatic pumps. Thus, it can be incorporated in autonomous micro- and nanorobotic approaches presented so far^69^. It should be noted that the potential applied here is sufficiently small compared to the potentials where electrolysis of water is taking place (*E* =  + 1.23 V vs SHE cf. ref.^70^) Hence, no development of gas bubbles is observed, neither for the flat electrodes nor for electrochemical grippers.

Currently, micro- and nanomanipulation by AFM is based practically exclusively on applying lateral forces with the tip and ‘pushing’ or ‘dragging’ the objects over the substrate^3^. The limitations of this established approach are evident: On the one hand the danger of mechanical damage and on the other hand the restriction to two-dimensional structures. By contrast, the electrochemical gripper allows not only for overcoming these limitations but is also a much more universal approach; It can be scaled down easily and could be also merged with other approaches from robotics, such as autonomous robots or soft robots. By ‘grabbing’ an object, placing it at a defined position, and then ‘releasing’ it, no shear forces are applied. Such forces are known to destroy samples, in particular soft, biological ones. Additionally, ‘gripping’ evades the central problem of push-manipulation: During the process, the object, which has to be manipulated must remain in contact with the substrate. Hence, extending structures into the third dimension or manipulation on rough substrates is intrinsically problematic to impossible. Moreover, the technique does not allow to separate objects that are attached to each other by van der Waals forces, e.g. colloidal particles. The same would be valid if adhesive forces between particles are too strong to be overcome by a repulsive force due to the externally applied potential. However, we did not encounter these situations normally in our manipulation experiments that concentrated on the preparation of 2D-structures. In the case of 3D-structures these situation would be encountered to a much higher degree.

Another important advantage of the electrochemical grippers is the possibility of merging them with existing algorithms from AFM-based nanomanipulation and macro/micro-robotics^20,71–73^. Commonly, AFM-based nanomanipulation is founded on laterally pushing a particle or object by applying shear forces. Imaging and manipulation are both based on AFM^8^. By applying different potentials it would be possible to pick and place the particle directly, while imaging would still be possible with the potentials leading to repulsive interactions. In the case of coupling electrochemical grippers with an AFM, the accuracy of the positioning is given by the lateral resolution of the piezo-scanners, which is on the Angstrom level. However, in combination with coarse positioning by stepper motors the range is practically limited only by the dimension of the liquid cell. If the particles are large enough to be identified by optical microscopy, algorithms utilized by automated micromanipulation in combination with optical or electron microscopy can applied directly^74–77^.

What are the fundamental limitations of electrochemical grippers? Firstly, such grippers will only work in liquid media, mainly electrolytes, in order to guarantee potentiostatic control and suppress capillary forces. However, for biomedical applications and most cases of nanofabrication, especially with soft materials or nanoparticles, the liquid phase is not a limitation but a requirement. One limiting factor for biological samples would be surface fouling. To prevent this, especially in high ionic strength solutions, an antifouling agent, like an amphifunctional thiol-SAM, could be incorporated into the gripper^78–80^. Additionally, the gripper electrode could be cleaned by cyclic voltammtry as it can be performed for flat electrodes^81^. High ionic strengths of electrolyte solution will lead to smaller forces due to reduced diffuse layer overlap^41,63,64^. However, increased viscosity would not represent a problem^36^. Hence, also manipulation in ionic liquids would be possible, provided that an electrochemical control of the gripper electrode can be ensured. Secondly, there is only a certain size range for the objects that can be handled: Too small, then the van der Waals forces would dominate; too big, the surface forces cannot compensate for gravitational effects. Essentially, the diffuse layer overlap should allow for tuning the overall interaction, which is possible for a wide range of adhesion forces due to hydrophobicity. For hard colloidal particles the size range starts approximately at 50–100 nm and goes up to 5–8 μm, also depending on their surface charge, roughness, and density, respectively. However, for larger particles conventional mechanical grippers would be most-likely a more convenient method for manipulation. Thus, the here-presented approach of electrochemical grippers allows to bridge the gap between objects of the micro- and the nanoscale for two-dimensional manipulations. Objects smaller than 100 nm might be handled, but low van der Waals forces would be necessary, which requires small Hamaker constants. Luckily, materials that fulfill these requirements would be the most interesting for robotics at this length scale: macromolecules, like proteins or lipids. For both, it has been reported that their adhesion to electrodes can be tuned and adsorption as well as desorption can be controlled depending on the externally applied potential^82,83^. However, with decreasing size of the objects, the geometry and dimensions of the tip become more important and would have to be adapted specifically^84,85^. In particular, as an AFM tip might be needed for manipulation as well as for imaging. In summary, a size range of colloidal particles of 0.7–8 μm would be readily accessible by the here-presented grippers. Most likely also very soft objects, such as hydrogel particles^86^ can be handled provided that the adhesion hysteresis is not too large.

In this respect, the possibility to ‘switch’ the charging state and thus the interaction forces may become very helpful to tune the tip for imaging without unintentionally disturbing and thus manipulating the objects. Moreover, the surface modification of the electrode and its roughness allow additional tuning of the interaction forces by tuning the contribution of solvent exclusion and the extent of diffuse layer overlap during contact as reported previously^41^. In difference to previously reported robotic grippers that are based on the electrochemical switching of a hydrogel coating^27^, the electrochemical grippers developed here are based on a direct manipulation of the interaction forces without an intermediate electrochemically active layer^27^. This additional layer provides the advantage of large adhesion forces and is, therefore, highly appropriate for macroscopic surfaces but is limited at smaller length scales due to the film morphology and thickness. At small length scales, the surface forces due to the diffuse layer are sufficient for manipulation. The lateral resolution of the positioning with electrochemical grippers is in principle only limited by the actuators. Here, the grippers have been implemented on an AFM-cantilever, thus the positioning resolution is in the sub-nanometre regime. Therefore, the main limitation is given by the dimension of the AFM-cantilever and the resolution of the optical microscope used to control the manipulation process. However, the here-presented grippers can also be used in combination with alternative actuation systems that are more suitable for soft robotics^14,15^.

## Methods

### Materials

Tipless gold-coated AFM-cantilevers used for the preparation of colloidal probes and electrochemical grippers were obtained commercially (CSC-37, Cr–Au coated from both sides, µmasch, Tallinn, Estonia). Cathodic insulating paint (Clearclad HSR, Clearclad Coatings Inc.) was used for the insulation of the electrochemical grippers. Silica particles with a nominal average diameter of 6.8 µm (Bangs Laboratories Inc.) were used to prepare colloidal probes for direct force measurements and for manipulation. UV curing adhesive (NOA63) was purchased from Norland products. Red insulating resist was purchased from GC Waldom. All aqueous solutions have been prepared with deionized water of Milli-Q grade (resistivity > 18 mΩ cm^−1^, Merck Millipore, Darmstadt, Germany). Ionic strength and pH of the solutions were adjusted to pH 4 and ionic strength of 0.1 mM using 1 M HCl (Titrisol, Merck, Darmstadt, Germany). All solutions were degassed for at least 60 min before the experiments and filtered using a syringe filter with a pore size of 0.22 µm (Rotilabo, Carl Roth, Karlsruhe, Germany). Methoxy(dimethyl)octylsilane, ferrocyanide, ferricyanide, 11-mercapto-1-undecanol, chloroform, potassium nitrate were purchased from Sigma Aldrich. Hellmanex III was purchased from Hellma (Mühlheim, Germany). Silver wires insulated with polyimide and a diameter of 0.125 mm were purchased from Advent (Advent research materials, Oxford, England). Ethanol of HPLC grade was purchased from Carl Roth (Carl Roth, Karlsruhe, Germany).

### Preparation of electrochemical grippers and cyclic voltammetry

Gold-coated AFM-cantilevers were cleaned by dipping in ethanol and chloroform, followed by subsequent air plasma treatment (Zepto, Diener electronic, Ebhausen, Germany) for 30 min. An additional gold layer (99.99%) with a thickness of at least 100 nm was evaporated onto the cantilevers using a tectra minicoater (tectra, Frankfurt, Germany) to prevent complete removal of the gold in the focused ion beam (FIB) treatment. In order to contact the cantilever, a polyimide-insulated silver wire was connected to the cantilever chip by silver paint (G302, PLANO, Germany), fixated, and insulated using an UV-curing adhesive. Cathodic insulating paint was electrodeposited onto the cantilevers by applying − 3 V for 120 s in a 1:5 solution (v/v) of Clearclad-HSR and water. The electrodeposition was performed three times for each cantilever, with a rinsing step (water and ethanol) between each cycle. The insulated cantilevers were annealed at 180 °C for 1 h. The wire's contact was insulated further using insulating resist. For FIB-milling, a FEI SCIOS-FIB was used with a milling depth of 50 nm. All SEM images shown were also acquired at the same SEM.

FIB-milled cantilevers were cleaned afterwards by dipping them in ethanol and water, followed by UV-cleaning (Model 18, Jelight Inc.) for 10 min and subsequent dipping in ethanol. The FIB-milled cantilevers were then immersed into a 5 mM 11-mercapto-1-undecanol solution in ethanol for 1 h and rinsed with ethanol afterwards.

The spring constants have been determined by fitting the thermal noise spectra (Hutter-Bechhoefer method)^87^.

Cyclovoltammetric measurements were conducted in an aqueous solution of 5 mM ferrocyanide, 5 mM ferricyanide, and 100 mM KNO_3_ using a potentiostat (CH 750i, CH-Instruments)^49^. The scan rate used was 0.01 V/s.

### Surface modification

First, glass slides were cleaned using a 2% aqueous Hellmanex solution in an ultrasonic bath for 40 min at 40 °C, followed by 10 min air plasma treatment. The silane modification has been carried out by chemical vapor deposition with methoxy(dimethyl)octylsilane. The glass slides were put in a desiccator together with 30 µL methoxy(dimethyl)octylsilane. The desiccator was evaporated and placed in an oven at 90 °C for 20 min, 35 min, or 60 min, respectively. Static water contact angles for all substrates were determined by the sitting drop method (OCA-2O, Dataphysics, Filderstadt, Germany).

### Colloidal probe measurements on glass surfaces

Silica beads were glued onto tipless AFM cantilevers using UV-curable glue (NOA 63, Norland Adhesives) by means of a micromanipulator (DC-3 KS, Märzhäuser, Wetzlar, Germany). The micromanipulator was mounted on a fixed-stage microscope (Examiner, Zeiss, Oberkochen, Germany). The cantilever was cleaned beforehand by rinsing with ethanol and MQ-water, followed by 10 min plasma cleaning. For the gluing first, a small drop of glue with a diameter slightly less than the ones of colloidal particles has been placed on the cantilver. Then a freshly etched tungsten wire has been used to place a colloidal particle in the glue drop. Curing was carried out with the mercury lamp attached to the optical microscope. The procedure was similar to the one reported previously^41^. All force measurements were performed with a dedicated atomic force microscope (MFP 3D, Asylum Research, Abingdon, United Kingdom) mounted on an inverse optical microscope (Observer, Zeiss, Oberkochen, Germany). For determining the effective potential and regulation parameter *p* of the silica beads, 30 consecutive force curves were measured with a silica colloidal probe against silica beads glued to a glass substrate. The force curves were fitted using a homemade algorithm taking charge regulation into account^53^. For the measurement of substrate adhesion 30 force curves were measured with colloidal probes against the silane covered glass slides and bare glass slides, respectively. Adhesion forces were determined from the force-curves based on a custom written procedure in IGOR PRO (Wavemetrics) that determined the absolute minima in the force-curves upon retraction.

### Direct force measurements under potentiostatic control

For the potentiostatically controlled direct force measurements, silica-particles were glued onto a Hellmanex-clean glass slide using NOA 63 and a micromanipulator attached to an optical microscope (Examiner, Zeiss, Oberkochen, Germany). For the preparation of colloidal probes, first a small drop of UV-curing NOA 63 glue was picked up with an etched tungsten wire, and placed on the substrate. A silica particle, dried from aqueous solution onto a clean glass slide, was placed onto the drop with a clean micromanipulator needle. The placed bead was cured for 1 min with UV light. For the force measurements, 30 force curves were performed in an aqueous solution (ionic strength of 0.1 mM and pH 4). The working electrode was the electrochemical gripper electrode, a Pt-wire was used as counter electrode and a chlorinated Ag/AgCl wire was used as a pseudo reference electrode. The electrochemical cell has been controlled by the same potentiostat also used for CV-measurements. The half-cell potential of the pseudo-reference was controlled against a calomel electrode (RE2, BASi Inc.) in an aqueous solution with ionic strength of 0.1 mM and pH 4. Force deflection curves were averaged and evaluated using a homemade procedure. Approach and retraction curves have been baseline corrected individually.

### Particle manipulation by electrochemical grippers

Silica particles were sedimented on a plasma-clean glass slide in aqueous solution with ionic strength of 0.1 mM and pH 4. The electrochemical cell and potentiostat were the same as described for the potential dependent force measurements. After alignment using the optical microscope, a potential of *ϕ* =  + 726 mV vs. SCE was applied to the cantilever, and the cantilever was approached toward the bead using the z-piezo. After withdrawal, the particle stuck to the cantilever. XY-movement was done using micrometer screws. For particle placement, a potential of *ϕ* = − 474 mV vs. SCE was applied to the cantilever, and the cantilever was approached to the substrate using the z-piezo movement.

## Supplementary Information

Supplementary Video 1.

Supplementary Information 1.

## Data Availability

The data that support the findings of this study are available from the corresponding author upon reasonable request.
