# Supplementary material for: Electrochemical grippers based on the tuning of surface forces for applications in micro- and nanorobotics
Source: Sci Rep. 2023 May 16;13:7885. doi: 10.1038/s41598-023-33654-6 (PMC10188592; doi:10.1038/s41598-023-33654-6)
Supplement: Supplementary file 2 — Supplementary Information 1. [file 41598_2023_33654_MOESM2_ESM.pdf]

Supplementary Materials for

Electrochemical Grippers Based on the Tuning of Surface Forces for Applications in Micro- and Nanorobotics

A. Karg[1], V. Kuznetsov[1], N. Helfricht[1], M. Lippitz[2], G. Papastavrou[1]*

[1] Physical Chemistry II; University of Bayreuth, Universitätsstraße 30, 95440 Bayreuth, Germany.

[2] Experimental Physics III; University of Bayreuth, Universitätsstraße 30, 95440 Bayreuth, Germany

*Corresponding author. Email: georg.papastavrou@uni-bayreuth.de

S.1 Setup for electrochemical control

For direct force measurements under potentiostatic control, an electrochemical cell consisting of a pseudo Ag/AgCl reference electrode fabricated by electrochemically covering an PTFE-insulated Ag wire with an AgCl layer (AC1-01 Automatic Chlorider, NPI electronic GmbH) and a coiled PT-wire as counter electrode were used. Supplementary Fig. 1 shows photographic images of the AFM setup and the electrochemical cell.

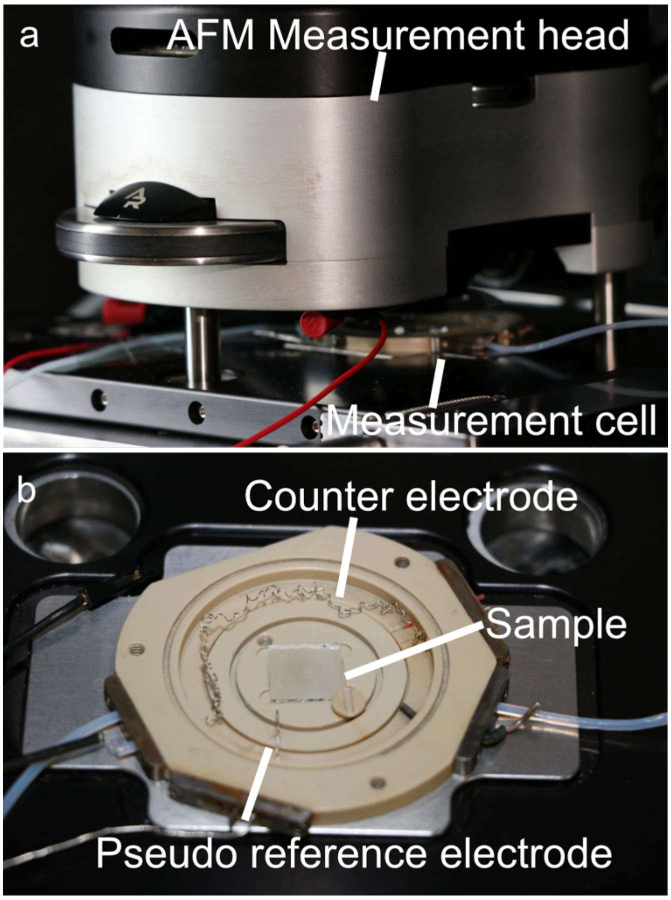

Supplementary Figure 1**. Setup for direct force measurements under potentiostatic control and manipulation with an electrochemical gripper.** (a) AFM head used for measurements in this work, with connected electrochemical measurement cell. (b) Electrochemistry cell for AFM measurements in liquid, with a chlorinated silver wire as pseudo reference and a Pt-wire as counter electrode. Here, a glass substrate is installed as sample.

S.2 Focused ion beam (FIB) milling depth

FIB-milling parameters for production of electrochemical grippers were determined experimentally by writing patterns with increasing FIB milling depth into the chip of an insulated cantilever. To determine the correct milling depth, namely just uncovering the gold surface while circumventing residual insulating film on the surface and preventing a complete removal of the gold, EDX and BSD images were used. As a result of this experiments, we chose a milling depth of 50 nm for preparation of electrochemical grippers.

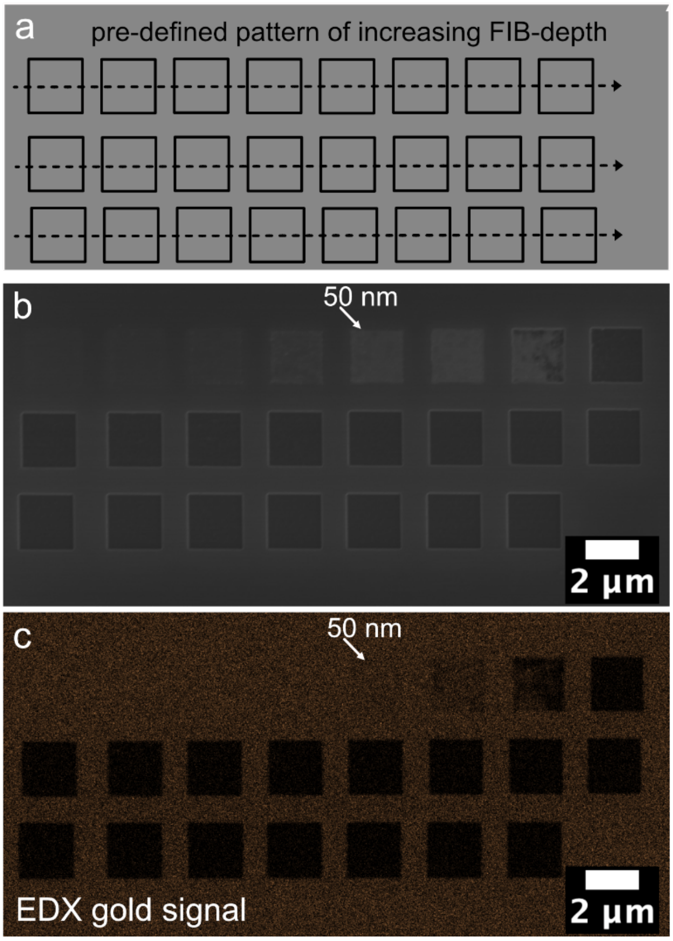

Supplementary Figure 2**. Focused ion beam (FIB) milling of an insulating film on a flat substrate.** (a) Schematic of a preliminary FIB-milling experiment. A pattern with FIB-milling depth increasing in 10 nm steps was milled into an insulated cantilever to find the ideal milling-depth. **(b)** SEM-image of FIB-milling tests. **(c)** Corresponding gold-EDX map. While the removal of insulating film is not visible because of the low thickness of the insulation film, a damaging of the gold is visible for FIB-depths that were too deep, like 70 nm in this case. The milling-depth that did not show any damaging of the gold layer, marked as “50 nm”, was chosen for electrochemical gripper preparation.

S.3 FIB-milling on AFM-cantilever

Using the parameters determined in S.2, a rectangular pattern was milled onto the free end of an insulated cantilever in several steps to exclude different behavior of insulating film on the chip and at the cantilever free end. An overview over a completely milled insulated cantilever and individual milling steps is shown in Supplementary Figure 3.

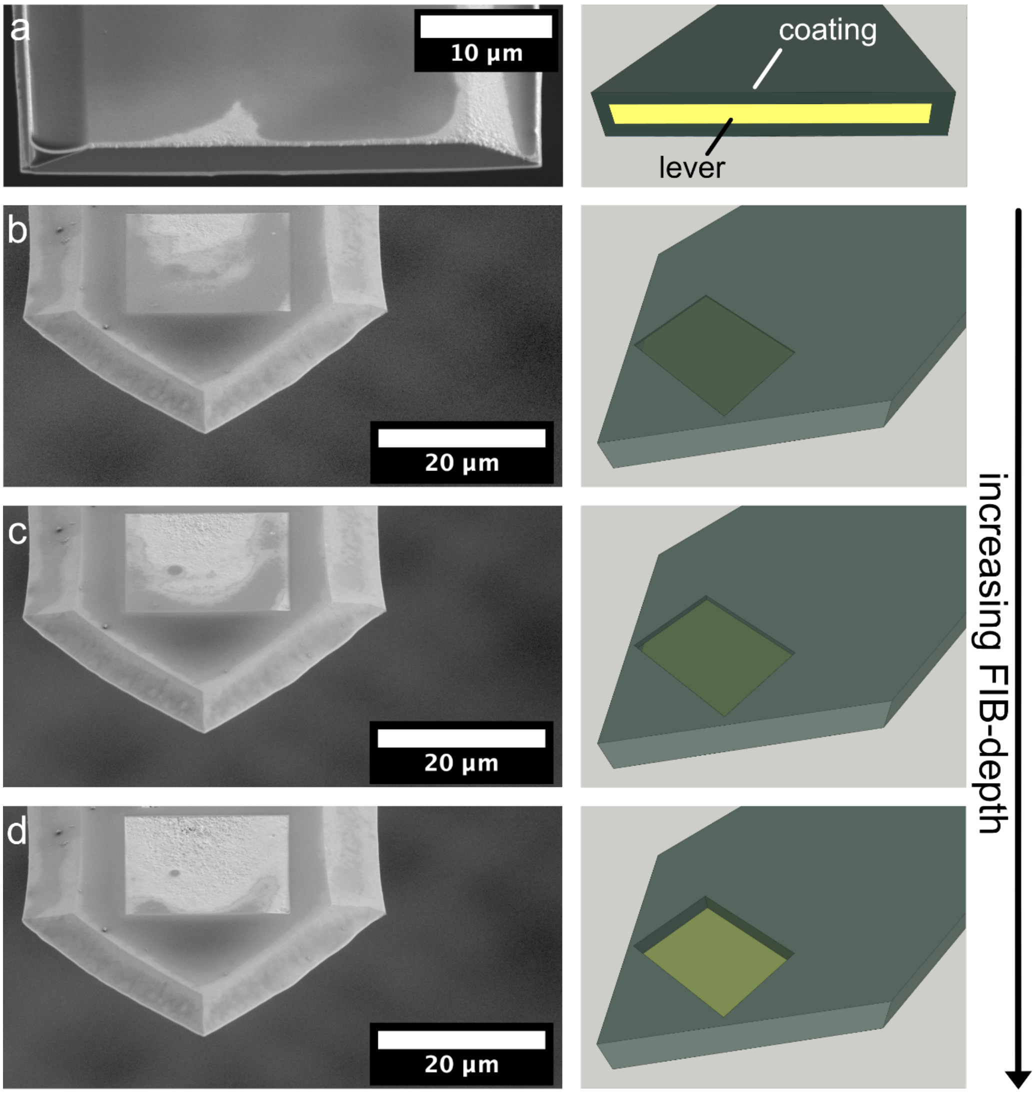

Supplementary Figure 3. **FIB milling of an electrode on an insulated cantilever.** **(a)** A cantilever that was cut through by FIB-milling to image the structure of the insulating film. **(b)** an initial FIB-milling depth of 30 nm in a rectangular shape. The non-insulated gold surface is visible brighter than the insulating film. **(c)** SEM image after another 10 nm FIB-milling step. **(d)** SEM-image after another subsequent 10 nm FIB-milling step, amounting to 50 nm total FIB milling depth.

S.4 Electrochemical characterization

To examine the electrochemical activity of electrochemical grippers, cyclovoltammetric experiments were done with both macroscopic electrodes and electrochemical grippers. The resulting cyclovoltammogramms are shown in Supplementary Fig. 4.

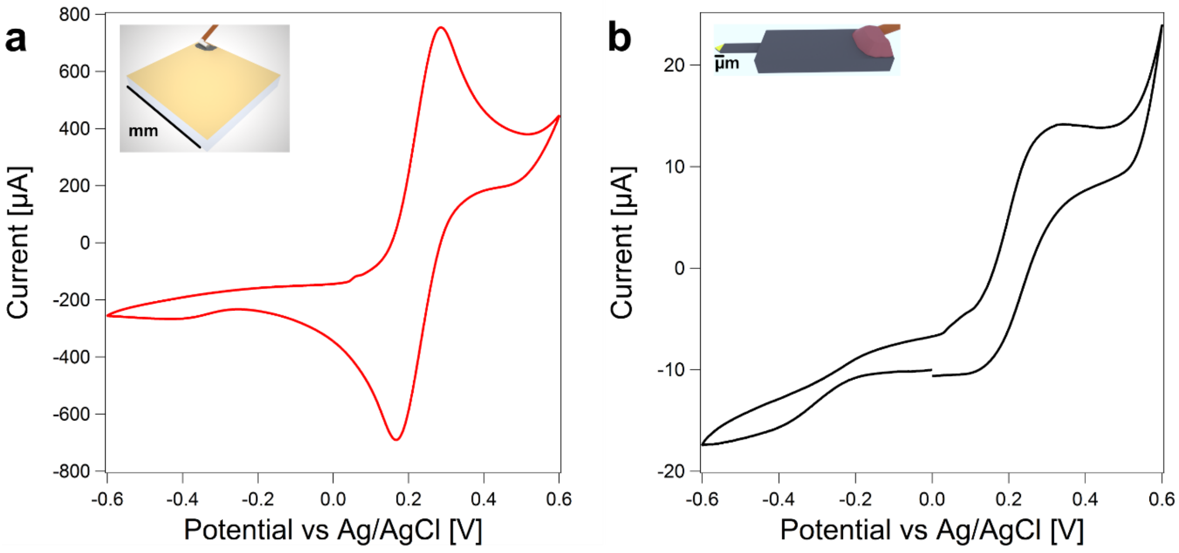

Supplementary Figure 4**. Electrochemical characterization by cyclic voltammetry (a)** Cyclovoltammetric measurement of a macroelectrode (11 mm x 11 mm) in aqueous solution of ferrocyanide (5 mM) /ferrocyanide (5 mM) and 0.1 M KNO_3 obtained at scanning speeds of 0.01 V/s. The current signal shows the isolated shapes at 0.3 V and 0.18 V expected for a macroelectrode. **(b)** Cyclovoltammetric measurement of a FIB- Cantilever electrode with an area in the µm-range. A superposition of isolated reduction and oxidation peaks onto the sigmoidal shape that is typical for the cyclovoltammogramm of a ultramicroelectrode is visible.

S.5 Potential-dependent force measurements

To determine the behavior of electrochemical grippers under potentiostatic control during force-measurements, Force-Distance curves on immobilized silica particles were measured using a potentiostatic controlled electrochemically gripper while applying different potentials on the free electrode at the cantilever end. The resulting force curves are shown in Supplementary Fig. 4. For all potentials higher than +136 mV, attractive behavior was visible, while for the more negative potentials repulsive behavior can be observed.

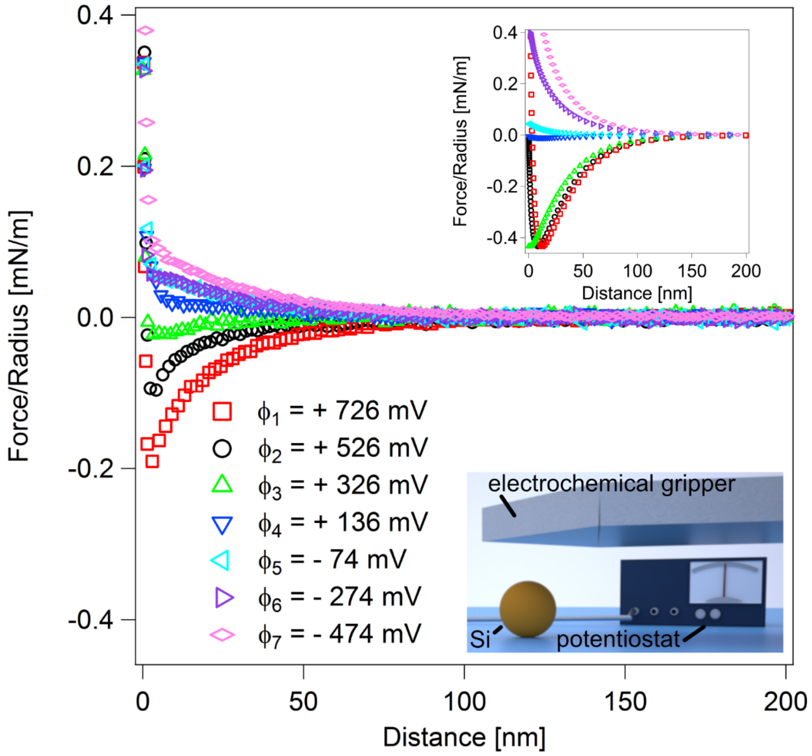

Supplementary Figure 5**. Force versus distance profiles for different applied potentials.** Measured force vs distance curves of a cantilever electrode against an immobilized silica bead depending on the applied potential in aqueous solution (pH = 4, I = 0.1 mM). All potentials shown were normalized against a SCE electrode. For a high negative potential, the curve is completely repulsive. The more positive the applied potential gets, the less repulsive is the force curve. From +326 mV vs SCE, an attractive interaction can be observed, that gets more attractive for more positive applied potentials. The inset shows force curves that were calculated using the Poisson-Boltzmann equation and consideration of charge regulation. As values for the effective potentials of the electrode, literature values were used (*1*). The effective potential and regulation parameter for the silica beads was determined by symmetric measurements (cf. Fig. S6).

S.6 Poisson-Boltzmann theory

To further analyze the behavior of the electrochemical grippers at the potentials used for manipulation, their force profiles were fitted to the full Poisson-Boltzmann equation taking into account charge regulation. The resulting fits are shown in Supplementary Fig. 6.

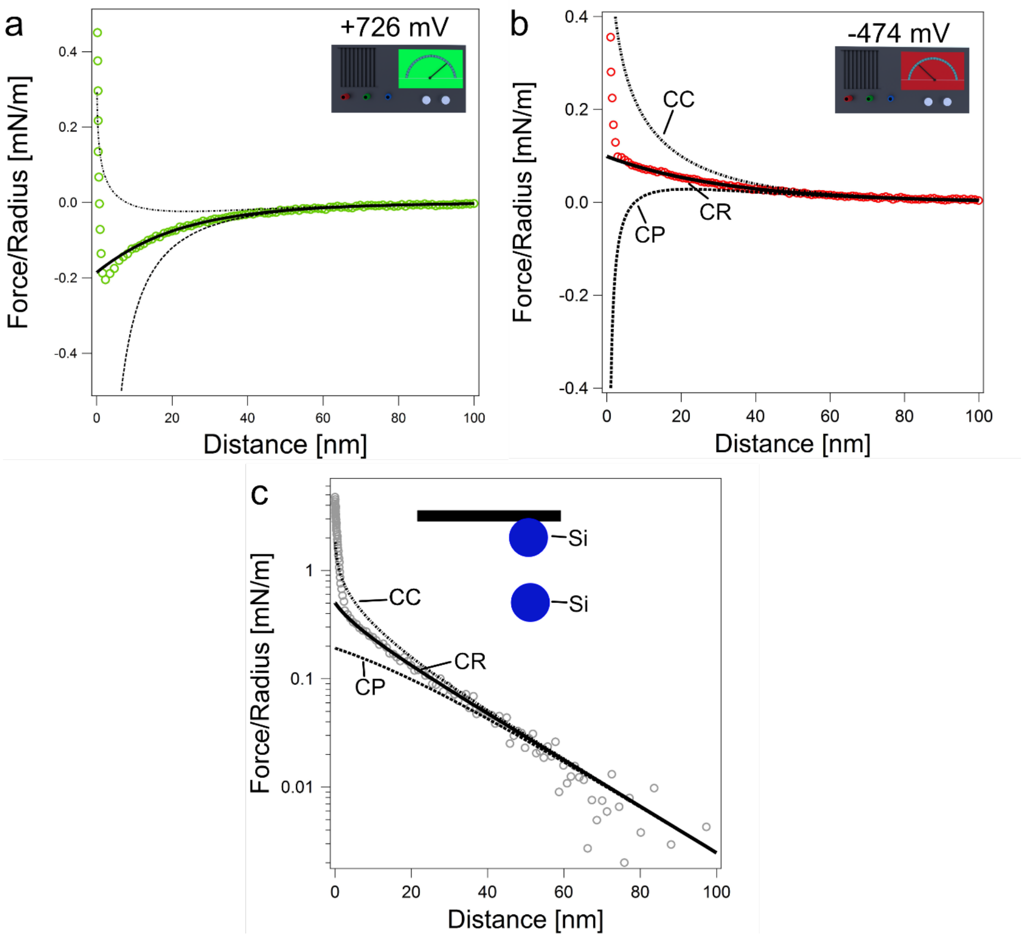

Supplementary Figure 6**. Fitting the force profiles to the full solutions of the Poisson-Boltzmann equation including charge regulation. (a)** PB-fit with consideration of charge regulation of a force curve of a cantilever electrode against an immobilized bead while applying a potential of 726 mV vs SCE, the potential that was also used for manipulation experiments in aqueous solution (pH = 4, I = 0.1 mM) Charge regulation takes into account the charge regulation parameter p, which assumes values in a range between p=0 and p=1. The dashed lines indicate the classical boundary conditions needed for a solution of the Poisson-Boltzmann equation. The boundary condition for p=0 is the constant potential (CP) condition, indicating a constant diffuse layer potential for all separations. Meanwhile, p=1 corresponds to the constant charge (CC) condition, indicating a layer of ions is adhering to the surface, and therefore leading to a constant charge density over all separations. The solid line represents fits to the constant regulation (CR) approximation, which accounts for charge regulation between two surfaces. For small distances, the CR approximation allows for a good description of interaction forces. (*2*, *3*) **(b)** PB Fit of a force curve with an applied potential of -474 mV vs SCE for the analogous experiment. **(c)** PB-Fit for the measurement of a colloidal probe against an immobilized silica particle to determine the effective potential Ψ and regulation parameter

p of a silica colloidal probe. The effective potential was determined to be Ψ = -24 mV, while the regulation parameter was determined to p=0.92

S.7 Comparison of electrochemical gripper and flat electrode

The effective potentials for the electrochemical grippers used in this work were determined by PB-fitting as shown in Supplementary Fig. 6 for all potentials used in S.5. Supplementary Fig. 7 shows a comparison of these potentials with the potentials on a flat electrode determined by Kuznetsov et al . (*1*).

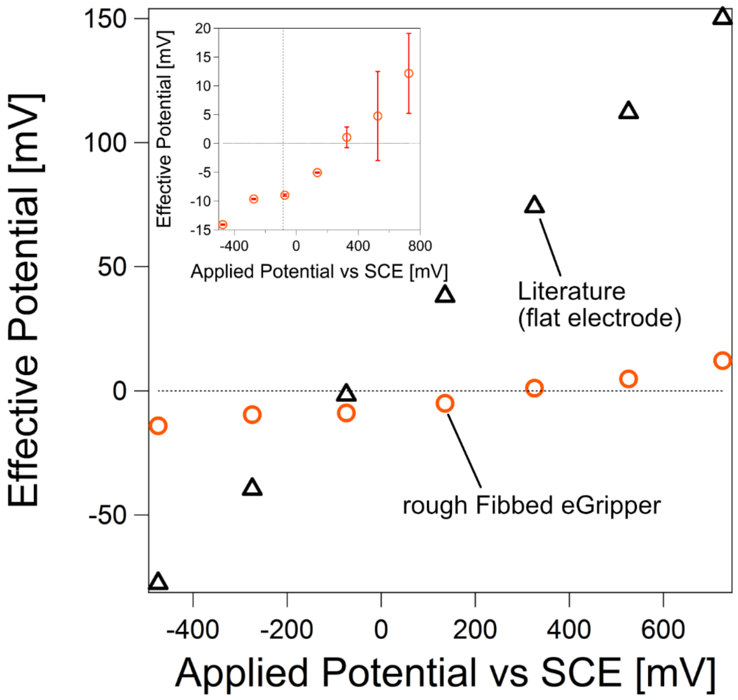

Supplementary Figure 7**. Diffuse layer potentials as function of the applied potential to the electrochemical gripper.** The effective potentials determined by PB fitting (cf. Fig. S6) are shown as a function of the applied potentials. The inset shows a close-up of the data obtained in this work. The dotted line shows the potential of zero charge from Kuznetsov et al. (*1*).

The effective potentials from this work for FIB-milled electrochemical grippers are compared with literature values of Kuznetsov et al. (*1*), who did a similar experiment for colloidal probe vs ultra-flat gold electrodes with applied potential. The slope of the values is lower for the electrode cantilevers. We blame this disparity on the roughness of the electrode (cf. Fig. S9), since during the measurement parts of the silica colloids could not have been in contact with the electrode of the electrochemical gripper as a result of the high roughness.

S.8 Adhesion on electrochemical gripper

The pull-off force of electrochemical grippers against immobilised silica particles were determined for various applied potentials from force-distance curves (cf. Supplementary Fig. 5). Supplementary Fig. 8 shows a comparison with an inverted setup- a standard colloidal probe against a flat electrode under potentiostatic control and their respective surface topography.

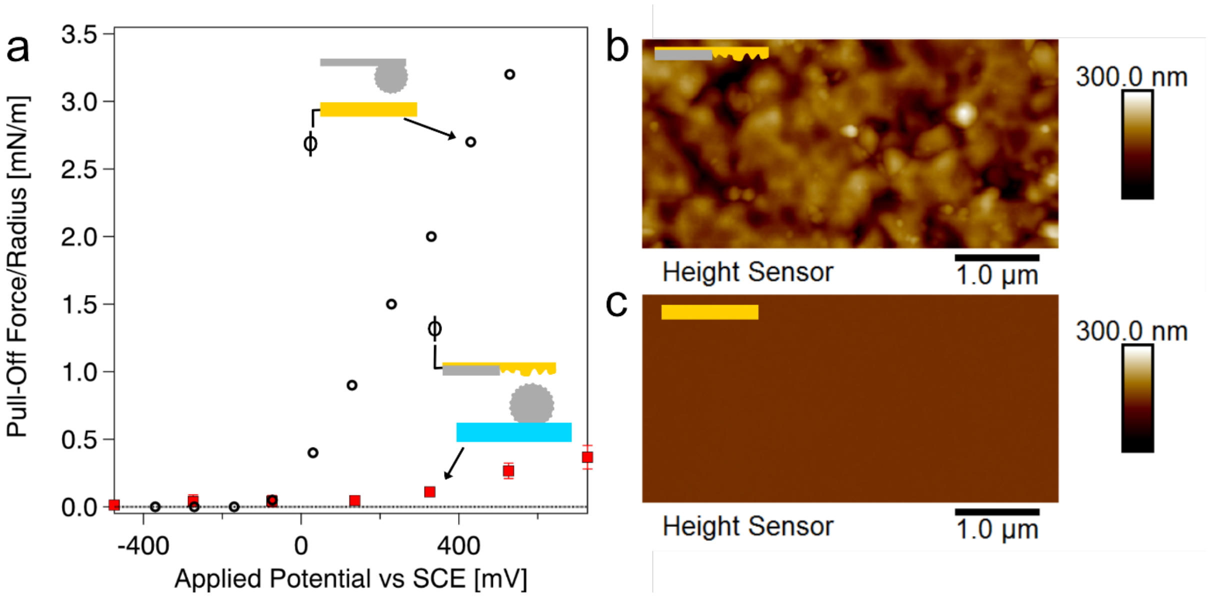

Supplementary Figure 8**. Adhesion between silica particles and the electrochemical gripper. (a)** The pull-off force (the absolute value of the adhesion force F_{Adh}) with a OH-terminated microelectrode cantilever against an immobilized silica bead is shown depending on the applied potential (red). For comparison, the pull-off force from for an inverse system, where the cantilever is immobilized on the cantilever as colloidal probe and measured against a ultraflat gold lectrode modified with OH-silane. The microelectrode cantilever shows a much lower pull-off force even for high attractive applied potentials. A possible reason for this is the high roughness of the microelectrode cantilever (cf. Fig. S9). **(b)** AFM topography image of electrochemical gripper (top) and ultraflat gold electrode (bottom), indicating the higher roughness of the FIB-milled electrochemical gripper.

S.9 Surface roughness comparison

We compared the surface roughnesses of FIB-milled electrodes, silica particles used in this study and template stripped gold electrodes used for adhesion experiments (cf. Fig. S-8). The resulting AFM topogrpahy images and profiles are shown in Supplementary Fig. 9.

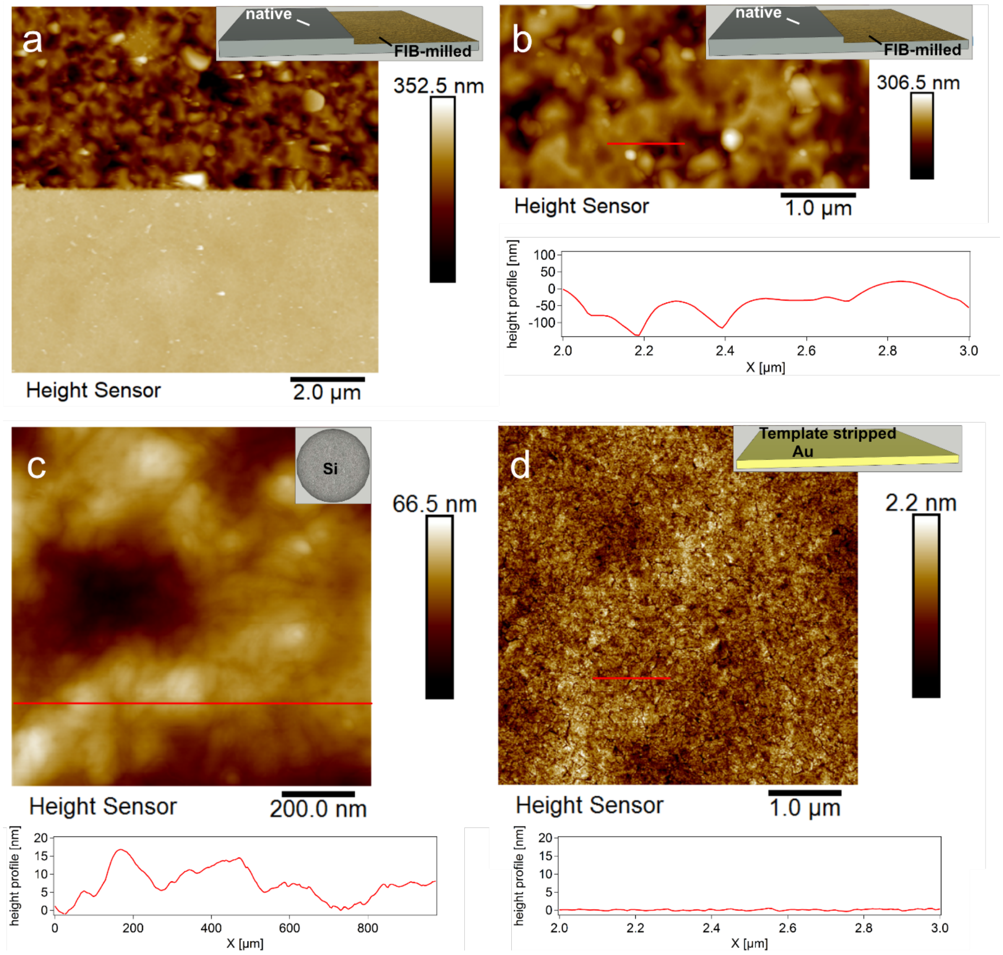

Supplementary Figure 9**. Comparison of surface roughness. (a)** Height image of the microelectrode cantilever as measured using a Bruker Icon AFM equipped with a OTESPA R3 cantilever in tapping mode. The smooth area on the bottom is the insulating film. The rough part on top is the FIB-milled gold area. **(b)** Topography of FIB-milled gold area and section through the height image. The position of the section is marked in the topography image. A roughness with valleys as low as 100 nm can be observed as result of the FIB-milling process. **(c)** Topography image of the silica beads used in this work, and section through the topography, showing the microroughness caused by Stöber-synthesis. These roughness decreases the adhesion compared to JKR theory. (*4*) **(d).** Topography of ultraflat gold electrode, as used in the work of Kuznetsov et al. (*1*) (cf. Fig S7). The same flat electrodes have been used to determine the pull-off forces for the inverse system (cf. Fig S8). An almost ideal flat surface is present.

While microroughness is most critical during adhesion (*4*), the high roughness of the electrochemical grippers could lead to a nonuniform part between the gripper and the particle,

explaining in part the inferior pull-off force compared to the inverse system (cf. Fig. 8), as well as the lower effective potential compared to literature (cf. Fig. 6).

S.10 Contact angle of silanized substrates

To determine the wettability of the silanized glass substrates used for adhesion experiment (cf. Fig. 4f), their contact angle was measured using sitting-drop method. The results are shown in Supplementary Table 1.

Supplementary Table 1. Contact angle measurements of methoxy(dimethyl)octylsilane (MDOS) -modified substrates**.**

**Silanization duration [min]**	**20 min**	**35 min**	**60 min**
**Pos. 1l**	49.3°	84.7°	104.9°
**Pos. 1r**	48.5°	84.5°	104.9°
**Pos. 2l**	48.4°	71.3°	97.6°
**Pos. 2r**	48.3°	69°	97.6°
**Pos. 3l**	46.6°	76.8°	100.5°
**Pos. 3r**	46.4°	76°	100.2°
**Avg.** $\theta \pm$ s.d.	47.9°± 1.2°	77.1°± 6.5°	101,0°± 3.3°

S.11 Movie of Manipulation experiments

We provide a sped-up movie (Supplementary Movie 'Electrochemical_Grippers.mp4' in mp4-format) showing the process of manipulating silica particles into complex structures by use of the electrochemical grippers presented in this work.

References

1. V. Kuznetsov, G. Papastavrou, Adhesion of Colloidal Particles on Modified Electrodes. *Langmuir* **28**, 16567-16579 (2012).
2. R. Pericet-Camara, G. Papastavrou, S. H. Behrens, M. Borkovec, Interaction between Charged Surfaces on the Poisson−Boltzmann Level: The Constant Regulation Approximation. *J. Phys. Chem. B* **108**, 19467-19475 (2004).
3. V. Kuznetsov, G. Papastavrou, Ion Adsorption on Modified Electrodes as Determined by Direct Force Measurements under Potentiostatic Control. *J. Phys. Chem. C* **118**, 2673-2685 (2014).
4. Y. I. Rabinovich, J. J. Adler, A. Ata, R. K. Singh, B. M. Moudgil, Adhesion between nanoscale rough surfaces: II. Measurement and comparison with theory. *J. Colloid. Interface Sci.* **232**, 17-24 (2000).
